# On the Specificity of Heparin/Heparan Sulfate Binding to Proteins. Anion-Binding Sites on Antithrombin and Thrombin Are Fundamentally Different

**DOI:** 10.1371/journal.pone.0048632

**Published:** 2012-11-12

**Authors:** Philip D. Mosier, Chandravel Krishnasamy, Glen E. Kellogg, Umesh R. Desai

**Affiliations:** Department of Medicinal Chemistry and Institute of Structural Biology and Drug Discovery, Virginia Commonwealth University, Richmond, Virginia, United States of America; University of Patras, Greece

## Abstract

**Background:**

The antithrombin–heparin/heparan sulfate (H/HS) and thrombin–H/HS interactions are recognized as prototypic specific and non-specific glycosaminoglycan (GAG)–protein interactions, respectively. The fundamental structural basis for the origin of specificity, or lack thereof, in these interactions remains unclear. The availability of multiple co-crystal structures facilitates a structural analysis that challenges the long-held belief that the GAG binding sites in antithrombin and thrombin are essentially similar with high solvent exposure and shallow surface characteristics.

**Methodology:**

Analyses of solvent accessibility and exposed surface areas, gyrational mobility, symmetry, cavity shape/size, conserved water molecules and crystallographic parameters were performed for 12 X-ray structures, which include 12 thrombin and 16 antithrombin chains. Novel calculations are described for gyrational mobility and prediction of water loci and conservation.

**Results:**

The solvent accessibilities and gyrational mobilities of arginines and lysines in the binding sites of the two proteins reveal sharp contrasts. The distribution of positive charges shows considerable asymmetry in antithrombin, but substantial symmetry for thrombin. Cavity analyses suggest the presence of a reasonably sized bifurcated cavity in antithrombin that facilitates a firm ‘hand-shake’ with H/HS, but with thrombin, a weaker ‘high-five’. Tightly bound water molecules were predicted to be localized in the pentasaccharide binding pocket of antithrombin, but absent in thrombin. Together, these differences in the binding sites explain the major H/HS recognition characteristics of the two prototypic proteins, thus affording an explanation of the specificity of binding. This provides a foundation for understanding specificity of interaction at an atomic level, which will greatly aid the design of natural or synthetic H/HS sequences that target proteins in a specific manner.

## Introduction

Heparin and heparan sulfate (H/HS) represent one of the four major classes of glycosaminoglycans (GAGs) that are being increasingly recognized as playing critical roles in many biological processes including hemostasis, growth and differentiation, immune response, and pathogen invasion [1], [2], [3], [4], [5]. Unlike other biological macromolecules, H/HS are linear polysaccharides biosynthesized in the absence of a template by utilizing only five different chain-modifying reactions following the assembly of a precursor heparosan. It is interesting that the 16 known isoforms of the enzymes involved in these modification steps, coupled with their spatial and temporal regulation, generate phenomenal structural micro-heterogeneity in the polymers [2], [5], [6].

Both H/HS are composed of alternating 1→4-linked uronic acid and glucosamine residues that are decorated with sulfate and *N*-acetyl groups. Theoretically, 96 different disaccharide sequences are possible for H/HS arising from uronic acid (UAp) residues that can bear either an –OH or a –OSO_3_– group at its 2- and 3-positions and glucosamine (GlcNp) residues that may contain either an –OH or –OSO_3_– group at its 3- and 6-positions as well as carry either an –NH_3_^+^, –NHSO_3_– or –NHAc group at its 2-position. However, to date, only 23 sequences have been identified in nature [7]. A back-of-the-envelope calculation shows that these 23 H/HS disaccharides can generate thousands of distinct sequences that may serve as domains for recognizing proteins. Further complicating this structural diversity is the conformational variability of the iduronic acid (IdoA*p*) residues, which exist in multiple forms of which ^1^C_4_ and ^2^S_O_ are usually preferred [8]. The combination of sequence and conformational possibilities results in arguably the most structurally diverse library that nature synthesizes using only a handful of substrates and reactions.

Despite this structural diversity, only one H/HS sequence has been found to recognize its target protein with high specificity. This sequence, the DEFGH pentasaccharide sequence that binds antithrombin [9], [10], satisfies specificity considerations from both the biological, i.e., how unique is the binding mode among many possible modes, as well as the chemical, i.e., how unique is the sequence among the many sequences, perspectives. The distinguishing feature of this sequence is the presence of the central 3-*O*-sulfated GlcN*p* residue, which occurs rarely in H/HS. Absence of this rare monosaccharide generates a major binding as well as functional defect. The GlcN*p*3S is also present in an octasaccharide that binds to glycoprotein D of herpes simplex virus-1, although it has not been ascertained as yet whether this is a high-specificity interaction [11], [12].

Several other H/HS sequences have been suggested to be specific, e.g., high-affinity sequences that recognize growth factors [5], [13]. Yet, whether these are indeed so is a matter of major debate, as a large number of fairly distinct H/HS sequences appear to bind the same protein with variable affinity [13], [14]. Phenotypic examples that support the possibility of specific or selective H/HS–protein interactions have been uncovered, e.g., renal agenesis arising from a lack of 2-*O*-sulfotransferase and Wnt signaling effects upon removal of 6-*O*-sulfate groups [5]. However, the pair of interacting partners remains unclear at present and hence it is difficult to assess and confirm molecular specificity as the basis of the phenotype.

At the other extreme of the antithrombin–H/HS interaction is the thrombin–H/HS interaction, which is recognized as a prototypic ‘non-specific’ GAG–protein interaction [15], [16], [17]. Characteristic features of this interaction include: 1) absence of thrombin-induced resolution of H/HS into high and low affinity fractions, 2) substantial affinity of thrombin for a number of different anionic molecules, e.g., H/HS, aptamers, and sucrose octasulfate [18], [19], and 3) detailed salt-dependence studies that conform to a non-specific binding model [17]. In fact, the structure of a thrombin–octasaccharide complex demonstrates two different binding geometries of H/HS within the same crystal [20]. Thus, the thrombin–H/HS interaction is non-specific both from the biological and chemical perspective.

A central question of major importance to developing modulators of physiologic and pathologic processes is the specificity of H/HS interactions with proteins. In fact, because the fundamental structural basis for the origin of specificity remains unclear for protein–H/HS interactions, major difficulties arise in designing H/HS molecules that specifically target and modulate a protein. On the H/HS front, addressing specificity has been challenging. Development of preparatively homogeneous and structurally diverse libraries of H/HS sequences has been difficult. A growing trend has been to use high-resolution mass spectrometry [21], [22] and microarrays [23], [24] for identifying sequences that bind proteins. Computational approaches have also been used to elucidate high-affinity/high-specificity sequences for antithrombin [25], fibroblast growth factors [26], [27] and chemokines [28]. From the target protein perspective, several linear peptide binding motifs have been proposed as structural necessities for a unique recognition mode [29], [30]. Alternatively, a spatial distance relationship may be important [30], [31]. Recently, a ‘CPC’ (cation–polar–cation) motif has found to be commonly present in heparin-binding proteins [32]. These ‘rules’ will most likely be expanded, as recently some 435 human proteins have been identified to constitute the H/HS interactome [33].

A key requirement for engineering specificity from a drug design perspective is the development of spatially resolved and/or directional short-range forces such as van der Waals interactions and hydrogen bonds. The majority of H/HS–protein interactions rely upon long-range and non-directional Coulombic interactions, which have a 1/r distance-dependence – as compared to van der Waals forces with a 1/r^3^ to 1/r^6^ dependence. It is known that sulfate groups (–OSO_3_–) of H/HS can recognize arginines through the formation of directional, bidentate interactions [34], i.e., possessing both strong Coulombic and hydrogen bond components, and thus substantively enhancing binding energy. This implies that engineering specificity is possible through arginine – sulfate interaction. Yet, even though thrombin has at least five arginine residues in its heparin-binding site (HBS), its interaction is non-specific.

Beyond antithrombin–H/HS and thrombin–H/HS systems, no other protein–H/HS system has been studied extensively both in solution and in crystal form. Despite this limitation, understanding the differences in how antithrombin and thrombin recognize H/HS is expected to provide a template for specificity features that can drive interactions of H/HS. Thus, we developed a simple structure analysis approach to explore the differences in HBSs of these proteins. Computation of solvent accessibilities and gyrational mobilities of arginines and lysines in the HBSs of the two proteins and analysis of their crystallographic thermal B-factors reveal sharp contrasts. Evaluating the distribution of positive charges in the two proteins reveals considerable asymmetry in antithrombin in contrast to substantial symmetry in thrombin. Cavity detection techniques suggest that although both HBSs are surface exposed, there are subtle differences between the two that allow H/HS to form a ‘hand-shake’ with antithrombin, while interacting only in a more transient ‘high-five’ with thrombin. Furthermore, there are differences in the solvation of these pockets that differentially affect the energetics of binding. Cumulatively, these differences in the binding sites result in major differences in recognition of H/HS sequences, which help explain specificity of binding. The work presents a foundation for understanding specificity at an atomic level and will be of value in the design of natural or synthetic H/HS sequences that target proteins in a specific manner.

## Methods

### Computational Software/Hardware

SYBYL-X 1.3 (Tripos International, St. Louis, MO) was used for molecular visualization and for *in silico* structural manipulation. Statistical analyses reported herein were also performed using SYBYL-X and implemented using SYBYL Programming Language (SPL). Molecular modeling was performed on Intel Xeon- and AMD Opteron-based CentOS 5.5 Linux and Intel Xeon-based Mac OS-X 10.6 (Snow Leopard) MacPro graphical workstations.

### Antithrombin and Thrombin Coordinates

Crystal structures of antithrombin and thrombin co-crystallized with heparin or heparin-like fragments, obtained from the RCSB protein data bank (http://www.rcsb.org/pdb/), were used to analyze intra- and intermolecular interactions (Table 1). Coordinates of antithrombin and thrombin from 1TB6 [35] and the ‘A’ and ‘B chains of 1XMN [20] were extracted and used for cavity analysis and prediction of bound water studies. The unresolved heavy atoms of Lys240 in 1TB6 and Lys236 in 1XMN were added and assigned an extended conformation. Hydrogen atoms were added to each protein with SYBYL-X 1.3.

**Table 1:** Crystal structures used in the analysis of the HBSs in thrombin and antithrombin.

PDB ID	Chain		Description	Res. (Å)	Missing residues	Ref.
	T*	AT*				
**1XMN**	AB		Thrombin–Heparin	1.85	K236	[20]
	CD				K236, K240	
	EF				R126	
	GH				K236	
**3B9F**	LH		T–Protein C Inhibitor–Heparin	1.60	K236, K240	[52]
**1E0F**	AD		Thrombin–Haemadin	3.10		[53]
	BE					
	CF					
**1JMO** †	LH		Thrombin–Heparin Cofactor II	2.20		[54]
**1TB6**	LH		Antithrombin–Thrombin–Heparin	2.50	K240	[35]
		I				
**2B5T**	AB		AT–T–H Mimetic (non-productive)	2.10		[55]
	CD					
		I			R132	
**1SR5**		A	AT–anhydrothrombin–H (mimetic)	3.27		[47]
**1T1F** ‡		A	Antithrombin (native)	2.75	R13, R47, K114, K125	[55]
		B			R13, R47, K114, K125	
		C			R13, R47, K114, K125	
**1AZX**		I	AT (active)–Pentasaccharide	2.90		[10]
		L	Antithrombin (latent)–Pentasaccharide			
**1E03**		I	α-Antithrombin–Pentasaccharide	2.90	R13, K125	[56]
		L				
**1NQ9**		I	AT (Intermediate)–Heparin	2.60	K11, R46, K125, R132	[57]
		L			R13, R46, R132	
**2GD4**		I	AT–S195A Factor Xa–Pentasaccharide	3.30		[58]
		C				
**3EVJ**		I	AT (Intermediate)–Pentasaccharide	3.00	K11, R46, K125, R132	[59]
		L			K11, R13, R46, R129, R132	

* Represents thrombin (T) and antithrombin (AT).

† 1JMO is not included in the calculation of radius of gyration, an outlier that is not bound to GAG.

‡ 1T1F is not included in the calculation of radius of gyration (*R_g_*), an outlier that has incompletely built important amino acids including R47, K114 and K125 and is not an activated form of antithrombin.

The B-factors, which represent in part the thermal motion and potential disorder of atoms in an X-ray crystal structure, were analyzed for all side chain atoms in the structures of interest (Table 1). These can, thus, indicate regions or residues of a protein that have more conformational mobility or flexibility [36].

### Theoretical Background for Calculation of Radius of Gyration

The radius of gyration *R*_g_ is often used as a measure of the compactness of a group or cluster of points. To measure the radius of gyration of terminal units of lysines or arginines, a metric of positional variability, the center-of-mass (COM) of the set of *n* points with masses *m* is first calculated. The COM is the mass-averaged point in 3D space that indicates perfect balance among the cluster of masses. For masses that are equal, as is the case here, the COM is the mean position of the *n* individual point masses (Eq. 1):
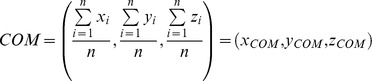



(The distance *r* between two points (*x_1_, y_1_, z_1_*) and (*x_2_, y_2_, z_2_*) is given by Eq. 2.
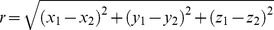



(The moment of inertia *I* of the set of masses rotating about the COM is the product of the mass and the square of the distance from the COM for each point (Eq. 3).
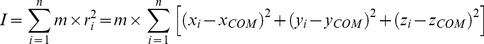



(The total mass *M* of the *n* points is *n×m* and if these points are distributed in a thin layer on the surface of a sphere, such that the moment of inertia *I* of the sphere is the same as that for the individual points, then the radius of gyration *R_g_* is the radius of this sphere is given by equation 4.
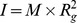



(4Rearranging Eq. 4, solving for *R_g_* and substituting for *I* and *M* yields Eq. 5, which shows that when each mass is equal, *R_g_* is the root-mean-square distance (RMSD) of the points from their COM.
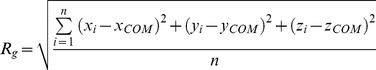
(5)


### Estimation of the Exposed Surface Area of Basic Residues

The MOLCAD functionality of SYBYL was used to generate a Fast Connolly surface for individual basic residues within the context of the HBS while taking into account neighboring residues; only the surface area that is exposed is included in the surface calculation. To generate a value for the maximal exposed surface area for each amino acid type, an analogous Connolly surface was generated for the central residue of a tripeptide Ala–X–Ala with an ideal α-helical backbone conformation. The percent exposure value for each basic residue was calculated by dividing the HBS exposed surface area by its maximal exposed surface area.

### Identification of Binding Pockets and Conserved Water Molecules

Binding pockets on the surface of antithrombin and thrombin were detected using the vectorial identification of cavity extents (VICE) algorithm [37] implemented in a local version of HINT [38] as a module within SYBYL. The VICE algorithm was used to search for pockets within the HBSs of thrombin and antithrombin (PDB ID = 1TB6). For antithrombin, the HBS was defined to include amino acid residues within 10 Å of the N^ζ^ (NZ) atom of Lys125, while for thrombin it was 15 Å from the N^ζ^ atom of Lys236. The grid resolution was set at 0.5 Å and the minimum closed contour value was set to be 60 Å^3^. The default cavity definition was set to 0.45 and the contour value was set to 0.4. All other variables were kept at their default values.

To investigate the extent of hydration, we used the binding site hydration algorithm of HINT [39]. In this approach, a grid-based algorithm combined with the HINT scoring function is used to identify the most probable locations of water molecules in the binding site. The HINT scoring function is atom-based and empirically parameterized and takes the form of equation 6.
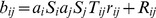
(6)In this equation, ‘*b*_ij_’ is the interaction score between atoms i and j, ‘*a*’ is the hydrophobic atom constant, ‘*S’* is the solvent-accessible surface area using a standard H_2_O probe, ‘*T*_ij_’ is a logic function that has a value of 1 or −1 depending on the nature of the interacting atoms (attractive or repulsive, respectively), ‘*r*_ij_’ is a function of the distance between atoms i and j (e^−r^) and ‘*R*_ij_’ is an implementation of the Lennard–Jones potential [38]. This formulation implicitly takes into account the entropic component of the free energy of binding of a small molecule, e.g., H_2_O, with a protein. It has been found empirically that about 500 HINT units correspond to 1 kcal/mol of binding free energy [38].

Water molecule placement was ‘focused’ in the pocket region, i.e., using the pre-computed cavity detection definition. The parameters for the water placement algorithm were set to ensure that the binding pocket was hydrated completely: the minimum water–protein distance was set to 3.0 Å, the van der Waals bump scalar was set to 1.02, the minimum H_2_O–H_2_O contact distance was set to 2.5 Å, and the minimum HINT score for placement of a water was set at −1000. An analysis of the relevance of each water molecule in the cavity was performed using the Water Rank and Score Report function of HINT, where Rank is a parameter encoding the quantity and quality of hydrogen bonds a water molecule may make [40]. An additional derived parameter, Relevance, correlates with water conservation [41].

## Results

Although a number of crystal structures for thrombin and antithrombin have been available for several years, a thorough and quantitative exploration of their heparin binding regions has not been performed up until now. In fact, the previous descriptions of these sites have been quite qualitative, e.g., “the size of the thrombin-binding site can even be as small as mono- or disaccharide fragment” [42]. By application of a number of unique computational structure analysis tools the characteristics of these HBSs are here described.

### Surface Exposure of Basic Residues Present in the HBS

The binding site of GAGs on proteins is usually considered to be surface-exposed and readily accessible [30]. This implies that the basic residues of the HBSs are generally assumed to be fully exposed to the bulk solvent. However, are all basic side chains equally exposed? More importantly, does surface exposure of the HBS residues vary significantly amongst heparin-binding proteins (HBPs), especially between antithrombin and thrombin?

The HBS of antithrombin consists of Lys11, Arg13, Arg46, Arg47, Lys114, Lys125, Arg129 and Arg132 residues, while in thrombin the basic residues are Arg93, Arg101, Arg126, Arg165, Arg233, Lys236 and Lys240. Of these, Lys114, Lys125 and Arg129 of antithrombin and Arg93, Arg101, Arg233, Lys236 and Lys240 of thrombin are important contributors to H/HS recognition [43], [44]. The exposed (water accessible) surface areas of each of these residues present in heparin co-crystal structures were calculated using the Fast Connolly surface generation algorithm. In this process, a sphere of 1.4 Å, which simulates a water molecule, is rolled on the protein surface and the area of contact for each residue measured. A tripeptide Ala–X–Ala, with X = Lys or Arg, was used as a control for 100% surface exposure.

**Figure 1:**
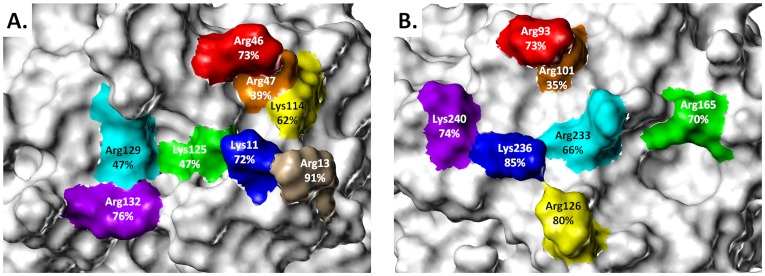
Relative solvent-exposed surface area for basic residues of the Heparin Binding Site: The SASA is calculated relative to a reference fully solvent-exposed residue present in a tripeptide. (A) Antithrombin's PBS (PDB ID = 1TB6). (B) Thrombin's exosite II (PDB ID = 1XMN, AB subunits). The exposed Connolly surface was calculated by rolling a sphere of 1.4 Å on the surface. See Methods for details.

Table 2 lists the relative exposure of individual basic residues present in the antithrombin pentasaccharide binding site (PBS) and thrombin exosite II. Figure 1 shows the values for antithrombin and thrombin mapped onto surfaces generated from 1TB6 and 1XMN, respectively. The surface exposure of the basic residues in the HBS of thrombin ranges from 66 to 85%, except for Arg101, which is 35%. In contrast, antithrombin's residues show a surface exposure range of 39 to 76%, except for Arg13, which displays 91%. Interestingly, only four of eight basic residues in antithrombin are predominantly surface exposed (exposure >2/3^rd^ of fully exposed), while for thrombin, the proportion rises to five out of seven. This simple analysis shows a fundamental difference between two apparently highly surface-exposed binding sites.

**Table 2:** Exposed surface area (SA) and radius of gyration (*R*_g_) of arginine and lysine residues in the pentasaccharide binding site of antithrombin and exosite II of thrombin.*

Amino Acid/Protein	Number of Observations†	Exposed SA ± S.D. (Å^2^)	% Exposure‡	*R*_g_ (Å^2^)	H-bond Partners
*Antithrombin*					
Lys11	10	92±1	72±1	2.19	—
Arg13	10	132±3	91±2	3.92	Asp14
Arg46	9	106±3	73±3	3.08	—
Arg47	13	56±3	39±2	0.32	Ser112, Thr115
Lys114	13	78±2	62±2	0.75	Phe122
Lys125	10	59±3	47±2	1.87	Asn45
Arg129	12	69±4	47±4	0.63	Thr44, Glu414
Arg132	8	110±4	76±2	3.46	—
*Thrombin*					
Arg93	11	105±2	73±1	2.52	—
Arg101	11	51±3	35±2	0.77	Asp100
Arg126	10	117±2	80±2	3.10	—
Arg165	11	102±3	70±2	0.52	Met180
Arg233	11	95±2	66±2	2.20	Asp178, Asn179
Lys236	7	108±3	85±2	3.29	—
Lys240	8	94±3	74±3	1.81	Gln244

* Exposed Surface Area was calculated using the Connolly surface area analysis, while *R*_g_ was calculated from the variation in the position of terminal group of Lys and Arg, as described in Methods.

† Represents the number of crystal structures used in calculations. This number is different for different residues because the number of completely resolved side chains varies among crystal structures.

‡ Calculated using fully exposed SAs for lysine and arginine in a tripeptide, which were found to be 127 and 145 Å^2^, respectively.

### Ease of Rotational Movement of Basic Residues Present in the HBS

The degree of surface exposure should directly correlate with side chain mobility, which can be expected to contribute to the specificity of interaction. First, we examined the trends in X-ray B-factor (thermal and disorder) for the relevant residues near the HBSs of thrombin and antithrombin. As expected, the mean B-factors increase with distance from the backbone along each chain, indicating greater thermal motion and or positional uncertainty for the polar end of the side chains. The B-factors are notably (up to ∼50%) larger for atoms in some side chains of the antithrombin structures (Lys11, Arg13, Arg46, Arg132) than in those atoms in thrombin structures. A large part of the difference may lie in the fact that the thrombin structures are of better resolution (mean 2.22 Å) than the antithrombin structures (mean 2.81 Å) and B-factors are expected to be better modeled with better quality (i.e., higher resolution) data.

The side chain mobility can be inferred from the observed variation in the position of a terminal atom in multiple crystal structures, which can be calculated as the radius of gyration (*R*_g_). In principle, *R*_g_ is the RMSD of a collection of entities of equal mass from their center of mass. Hence, 11 thrombin and 13 antithrombin structures (subunits counted individually) were aligned to thrombin monomer AB of 1XMN or antithrombin I monomer present in 1TB6, respectively (Table 2), and *R_g_* for basic residues was calculated using program scripts.

**Figure 2:**
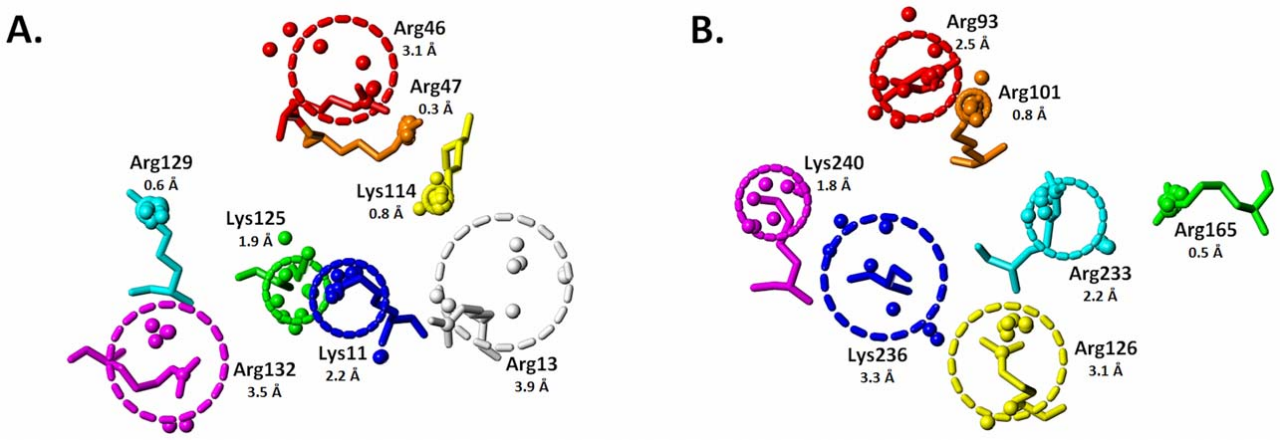
Radius (*R*_g_) of gyration for HBS basic residues: the HBSs of the pentasaccharide binding sites of (A) antithrombin and (B) exosite II of thrombin are depicted with gyrational mobility as thick dashed lines that convey the circumference of movement. The radius of gyration (Å) is listed below each basic residue. The basic side chains from (A) 1TB6 and (B) the AB subunits of 1XMN are shown. See text for details.

Figure 2 shows the observed variation in the position of the zeta heavy atom at the polar end of each lysine or arginine side chain superimposed on 1TB6 and 1XMN-AB structures. For antithrombin, Arg47, Lys114 and Arg129 displayed *R_g_* of 0.3, 0.8 and 0.6 Å, respectively, suggesting high spatial conservation across the series of crystal structures available in the literature (Table 2). On the other hand, Lys11 and Lys125 exhibit a modest level of spatial conservation with *R_g_* values of 2.2 and 1.9 Å, respectively, and Arg46 and Arg132 show a low degree of spatial conservation (*R*_g_ = 3.1 and 3.5 Å, respectively). Interestingly, Lys11 distributes into two distinct clusters, which may reflect a degree of spatial conservation.

In contrast, a majority of thrombin's basic residues including Arg93, Arg126 and Lys236 display *R_g_* higher than 2.5 Å (Table 2) indicating significant gyrational movement despite the presence of the bound H/HS. Arg233 and Lys240 display *R_g_* of 2.2 and 1.8 Å, respectively, which represent intermediate levels of gyrational flexibility. In a manner similar to Lys11 in antithrombin, Arg126 and Arg233 are distributed in two loci indicating a bimodal distribution. Finally, Arg101 and Arg165 of thrombin are most spatially conserved with *R_g_* of 0.8 and 0.5 Å, respectively.

Interestingly, a comparison of the mean zeta atom crystallographic B-factors with their corresponding *R_g_* values shows that two are modestly correlated for the examined basic residues of both antithrombin (r^2^ = 0.7) and thrombin (r^2^ = 0.4). This result was expected because lower *R_g_* results were computed for residues that have less positional uncertainty, while higher *R_g_* values were computed for residues that have more positional uncertainty. The *R_g_* analysis reveals that residues known to be important for H/HS recognition, especially for antithrombin (Arg47, Lys114, Lys125 and Arg129), are significantly less mobile than those known to be not important (Arg46 and Arg132).

**Figure 3:**
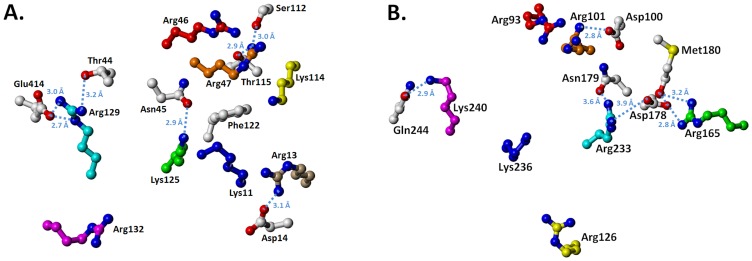
Analysis of neighboring groups for HBS residues that display reduced gyrational mobility: the basic side chains and neighboring amino acids from (A) antithrombin (1TB6) and (B) thrombin (AB subunits of 1XMN) are shown. Dotted lines indicate hydrogen-bonding and/or electrostatic interactions between neighboring residues. Inter-atomic distances (Å) are indicated for each polar interaction. Residues without neighboring interactions display high gyrational mobility. See text for details.

A counter argument to the above could be that the bound H/HS sequence induces reduction in gyrational motion. To assess whether this is the case, we compared structural differences around the amino acids with small and large *R_g_*. In the case of antithrombin, Arg47 bonds to Ser112 and Thr115, Lys125 interacts with Asn45, and Arg129 partners with Thr44 and Glu414 (Figure 3). On the other hand, Lys114 is held in place not because of a hydrogen-bonding partner but because of the hydrophobic influence of Phe122 and Pro12. An identical result is obtained with thrombin for less mobile residues. In this case, Arg101 forms a hydrogen bond to Asp100, Arg165 to Met180, and Lys240 to Gln244 (Figure 3). In contrast, residues displaying larger *R_g_*, e.g., Arg46 and Arg132 of antithrombin and Arg93, Arg126 and Lys236 of thrombin, tend to be unbonded and/or unengaged. Thus, the residues that are spatially conserved tend to have hydrogen-bonding partners within the binding site or have neighboring hydrophobic residues inducing fixed conformation at their Arg/Lys ‘stems’. This arrangement is the primary cause of significant reduction in the gyrational motion.

### Symmetry Elements Present in the HBS

**Figure 4:**
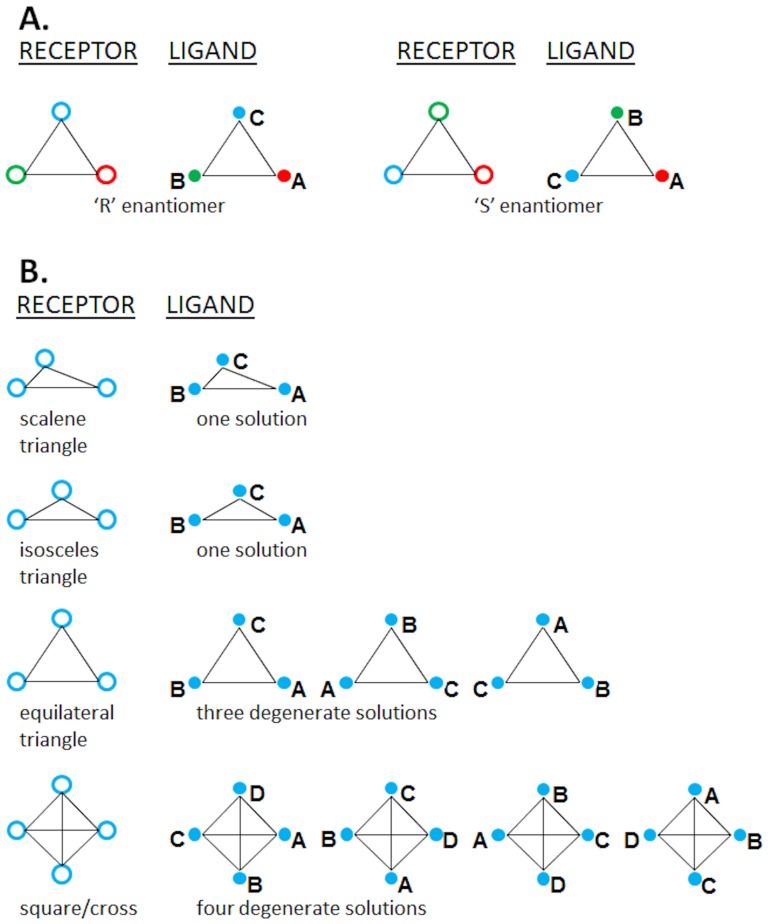
Two-dimensional symmetry elements in receptor-ligand interactions: (A) Traditional three-point concept of chiral ligand recognition with non-equivalent interacting pairs. (B) Conceptual representation of receptor–ligand interaction equivalence among receptor and ligand interacting groups with equivalent interacting pairs. Because the interacting pairs are equivalent, the spatial distribution determines the interaction specificity: the higher the degree of symmetry exhibited by the arrangement of interacting points in the receptor (e.g., basic side chains), the greater the number of ways in which a ligand containing a complementary set of interaction points (e.g., sulfate or carboxylate groups) can interact with the receptor.

Protein recognition of chiral ligands is highly stereo-specific, a property that arises from the intrinsic and complementary chirality of the binding site. A (+)-stereoisomer will not be effectively recognized by a binding site that prefers the (−)-isomer. The minimum number of unique elements necessary to engineer chiral recognition on a surface is three (see Figure 4). Thus, a HBS containing at least three basic residues should exhibit chiral, and hence stereospecific, recognition. In fact, stereo-specificity should generally increase as the number of basic residues increases because the binding site becomes more discriminatory and the number of possibilities that satisfy all interactions decrease. However, this expectation will be limited by the presence of symmetry elements (line, plane, etc.) within a binding site that can induce loss or reduction of intrinsic chirality, which may engineer a loss in recognition specificity.

**Figure 5:**
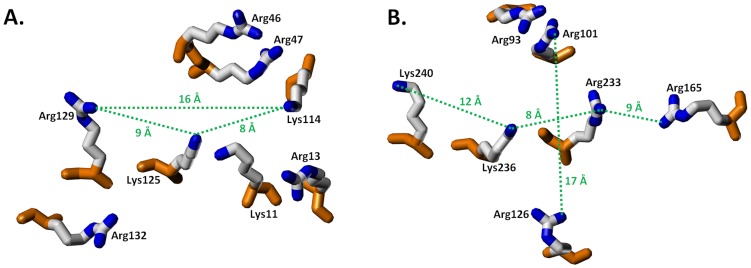
Symmetric elements identified among basic residues of HBSs of antithrombin and thrombin: (A) For antithrombin (1TB6), the three significant (in terms of H/HS binding) residues – Lys114, Lys125 and Arg129 – form a triangular geometry. (B) For thrombin (1XMN), the basic residues are arranged to form a ‘cross’ or ‘square planar’ geometry. See text for details.

An analysis of the HBS of antithrombin and thrombin reveals interesting symmetry-related differences. Figure 5 displays the arrangement of key basic residues at a two-dimensional level. For antithrombin, the three critical residues for H/HS recognition, i.e., Lys114, Lys125 and Arg129, are organized in a triangular manner. Other less important residues, e.g., Lys11, Arg13 and Arg47, introduce additional loci that can transform the triangular binding site into an asymmetric pentagon. In contrast, thrombin's seven important basic amino acids are organized along two lines/planes approximately perpendicular to each other. Considering their gyrational motion, Arg233 and Arg165 are located almost equidistant from Lys236 and Lys240, respectively. By the same token, Arg101 and Arg126 balance each other on the other axis (Figure 5). This geometric distribution of charges resembles a two-dimensional ‘cross’. Thus, the HBS of antithrombin carries an asymmetric distribution of important basic residues, while that of thrombin displays a significant reduction in asymmetry.

### HBS Cavity Analysis

To further elucidate the difference in the HBSs of antithrombin and thrombin, we focused on quantifying their width and depth. The cavity search algorithm VICE was developed utilizing the HINT (Hydropathic INTeraction) software toolkit [37]. VICE is a widely applicable algorithm that locates cavities, pockets, grooves, and channels on protein surfaces through an integer-based ray-tracing technique that detects the direction and extent of a cavity. The length, depth, volume, surface area and other cavity parameters are then calculated. VICE allows user-adjusted thresholds for specification of the minimum size of a cavity, its ‘cavityness’ as well as its putative location, which are particularly useful for identifying subtle differences between cavities.

**Figure 6:**
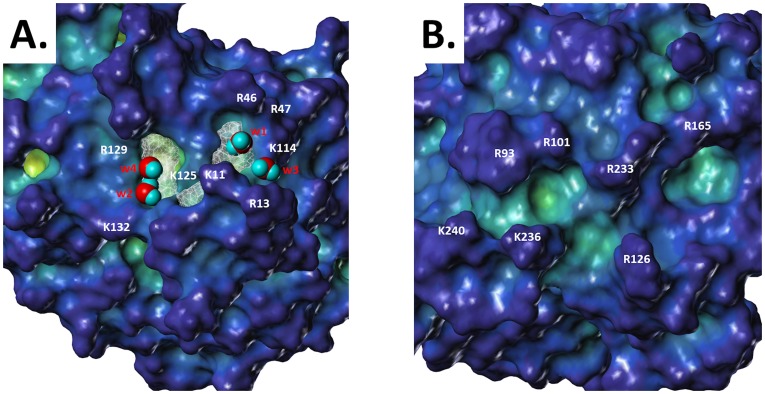
HINT-based detection of cavities and placement of water molecules: (A) In the antithrombin PBS, the detected cavity region is shown as a white mesh and the placed water molecules are shown with a space-filling representation. Four water molecules (*w1*, *w2*, *w3* and *w4*; space-filling representation colored by atom-type) are predicted to bind in this site when unliganded. (B) In thrombin exosite II, no deep cavity regions were identified using the specified VICE parameters (see methods section), although distinct grooves and shallow pockets are apparent. Surface color corresponds to cavity depth where blue indicates shallow regions and yellow indicates deeply buried regions. Figures were generated using the antithrombin–thrombin–heparin ternary complex (PDB ID = 1TB6). See text for details.

**Figure 7:**
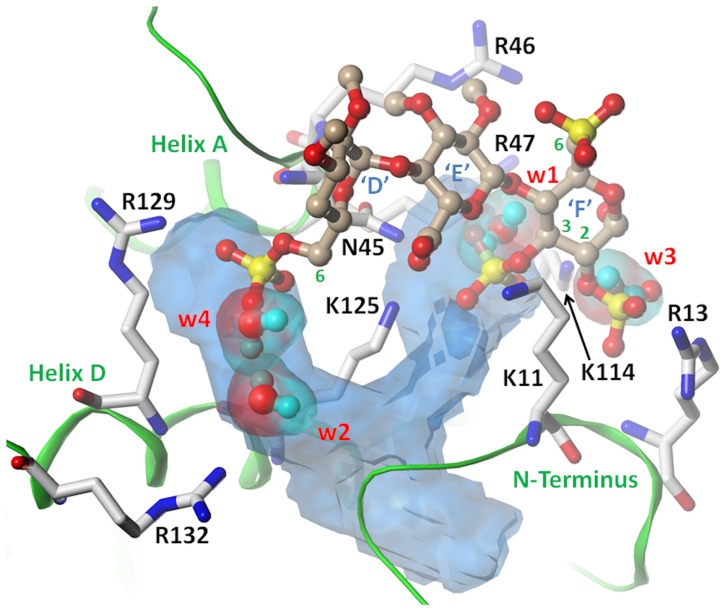
HINT-based hydration of the cavity in the PBS of antithrombin: A significant cavity is detected in the binding site (transparent blue surface) that is approximately 5–7 Å in depth and 15–20 Å in length. No such cavity was detected in thrombin (see figure 6). Four water molecules (*w1*, *w2*, *w3* and *w4*; ball-and-stick representation colored by atom-type) are predicted to bind in this site when unliganded. Co-crystallized pentasaccharide (only units ‘D’–‘F’ are shown; ‘G’ and ‘H’ are situated behind ‘F’ and are omitted here for clarity) is also shown in ball-and-stick rendering. See text for details.

Application of VICE to the HBSs of antithrombin and thrombin shows dramatic differences between the two. Whereas a reasonably sized, bifurcated, binding cavity was identified by VICE in the PBS of antithrombin, no such groove was identified in thrombin's exosite II. The identified cavity in antithrombin (Figure 6) is situated at the bottom of a groove that is flanked by helix A on one side and the *N*-terminus on the other. The pocket is largely hydrophobic in nature, but is bounded by basic residues Lys114, Lys125 and Arg129 of the D helix (Figure 7). The depth of the pocket ranges from 5 to 7 Å, while its length ranges from 15 to 20 Å. This implies that there is considerable cavity space available below the protein surface in antithrombin for a ligand to occupy.

Examination of the crystal structure reveals that these two pockets are occupied by 6-*O*-sulfate and 3-*O*-sulfate groups of residues D and F, respectively, of the high-affinity heparin pentasaccharide (Figures 6 and 7). Thus, certain sulfate groups of a saccharide sequence can interdigitate with Lys114, Lys125 and Arg129 of antithrombin. In an appropriate analogy, the H/HS–antithrombin interaction can be thought of as a firm ‘handshake’ between the two interacting complementary partners.

In contrast, the lack of a reasonably sized cavity in exosite II of thrombin does not allow inter-digitation of sulfate groups. This induces a more superficial interaction wherein basic residues of exosite II do interact with sulfate of heparin but without the formation of ‘more directional’ bonds. Biochemically, this characteristic becomes apparent as less non-ionic forces contributing to interaction, as noted by Olson et al. [17]. Thus, the thrombin-H/HS interaction is more analogous to a superficial ‘high five’.

### Prediction of Bound Water in the HBSs

Because charged residues bound it, the PBS cavity may reasonably be expected to be occupied by relatively tightly held (i.e., “ordered” or “relevant”) water molecules [41] in the absence of a ligand. Indeed, an analysis of high-resolution crystal structures has shown that such water molecules, presumably ordered, are found in surface grooves three times more often than anywhere else [45]. Displacement of such water molecules upon ligand binding provides an additional entropic driving force that supplements the enthalpic factors in the overall binding energetics. The expulsion of a single water molecule upon formation a protein–ligand complex can result in a change of −1.67 kcal mol^−1^ to ΔG^0^ [46] and the energy gain is additive if multiple water molecules are displaced.

There are a number of approaches to calculating the thermodynamic contribution of water to the ligand binding process [46]. We utilized tools within HINT [39], [40], [41] to predict the location of conserved water molecules in the aforementioned cavities. As these cavities will be occupied or occluded upon H/HS binding, such conserved water molecules may be ultimately displaced. Four water molecules, *w1*, *w2*, *w3*, and *w4*, were identified, as shown in Figure 6. Not surprisingly, three of these four water molecules, i.e., *w1*, *w3* and *w4*, were found to coincide with the locations of the three sulfate groups of heparin pentasaccharide (2S_F_, 3S_F_ and 6S_D_, subscripts indicate the residue). Table 3 lists the Relevance [41] and Rank [40] for these water molecules. Waters *w1* and *w2* display a Rank of 1.9 and 2.1, respectively, while *w3* and *w4* show a Rank of 0.9 and 0.0, respectively. This implies that, based only on the cavity's properties (and not those of other waters), *w1* and *w2* are highly likely to be present in the unliganded binding cavity, *w3* is marginally likely and *w4* is not very likely to be present. This analysis purposefully ignores the hydrogen bonding capabilities of solvation shell and/or bulk water because such contributions are less likely to induce an entropic boost upon H_2_O displacement to bulk. The Relevance and Rank values are also not high when the cavity floor is largely hydrophobic, which is especially the case near *w4*. While numerous waters are found in high-resolution crystal structures near hydrophobic surfaces, which suggests that they have a thermodynamic role [47], that role is probably to facilitate interaction through a low-cost displacement. Thus, the penetration of antithrombin's site by sulfate groups of H/HS is expected to result in replacement of 3 to 4 bound water molecules, which could help generate energy to the extent of as much as −5.0 kcal mol^−1^. This greatly supports the formation of a high specificity H/HS–antithrombin interaction, but the absence of a reasonably sized and similarly hydrated cavity in exosite II of thrombin suggests that it will not realize such energetic gain.

**Table 3:** Calculated HINT characteristics of the water molecules in the binding site water array [42].

Monomer Name	TOTAL for water*			Probability†		Weighting†		Relevance Prediction‡
	Rank	Score	Relevance	Rank	Score	Rank	Score	
HOH1	1.863	44.1	0.357	0.504	0.297	−0.064	−0.658	non-conserved
HOH2	2.058	57.0	0.390	0.551	0.320	0.103	−0.658	non-conserved
HOH3	0.902	−74.2	0.174	0.244	0.072	−1.000	−0.658	non-conserved
HOH4	0.000	−79.5	−0.040	−0.040	0.061	−21.136	−0.658	non-conserved
Mean	1.206	−13.2	0.221					

* Total Rank, HINT score and Relevance for water with respect to the protein.

† The Probabilities and Weightings are components of the empirical Bayesian-like Relevance equation – see reference 40.

‡ The Relevance model is built on the premise [41] that Relevance ≥0.50 represents the characteristics of a highly conserved water.

## Discussion

A cursory look at the pentasaccharide binding site of antithrombin and exosite II of thrombin reveals much similarity. Both are apparently surface exposed with no obvious deep pockets or long grooves, features on protein surfaces that traditionally are required for ligand binding domains. Both sites are composed of multiple, highly polarized basic residues and are flush with numerous solvent molecules. Both sites are extensive and span a large cross-sectional area of some 400 Å^2^, which is several-fold larger than that typically used by traditional, small drug-like molecules [48]. Yet, these similarities hide a glaring difference. The PBS of antithrombin preferentially recognizes a single H/HS structure, while exosite II of thrombin recognizes numerous heparin-like structures equally well. Understanding the foundation of this specificity, or lack thereof, is important.

Our work shows that the two H/HS binding sites display subtle, but important, differences in architecture. Even though one would expect side chains of lysine and arginine to be fully exposed, several residues of the HBSs of the two proteins are not. Arg47, Lys114, Lys125, and Arg129 of antithrombin and Arg101 of thrombin belong to this category (Table 2). Despite their reduced exposure, these residues are important for H/HS interaction [44], [49]. Interestingly, one of these residues, Lys125 of antithrombin, is involved in the initial recognition of heparin pentasaccharide [50], which in principle could be better served by greater extension and exposure of its side chain. Although Arg101 of thrombin has been implicated in H/HS binding, its importance is thought to be less than that of Arg236 and others [20], which were found to be essentially fully solvent exposed (Table 2). Thus, despite an apparent similarity, antithrombin and thrombin display an inverse relationship between the degree of residue burial and importance in H/HS binding.

Radius of gyration calculation reveals that the more buried residues are also generally less mobile. This is not too surprising because the methylenic groups of Lys and Arg introduce significant gyrational motion, which can be become pronounced upon enhanced surface exposure. This gyrational motion can be both advantageous as well as detrimental. A high gyrational sweep of Lys and Arg residues can more effectively serve as a ‘bait’ to attract anionic group(s) on H/HS from considerable distances and irrespective of the angle of approach. The non-directional and long-range Coulombic forces contribute to this process, resulting in an enhanced probability of interaction. However, too much gyrational motion can also be detrimental because it disfavors the formation of a strong, stable interaction, e.g., specific hydrogen bonds. Thus, buried residues with reduced gyrational motion are likely to engineer specificity of interaction.

In fact, residues known to contribute to specificity of the H/HS–antithrombin interaction, i.e., Arg47, Arg129 and Lys114, do display low *R*_g_ (Figure 2, Table 2). The only oddity appears to be Lys125, which is buried and critical for heparin binding, but displays intermediate mobility with a *R*_g_ of 1.9. It appears that this intermediate flexibility helps support its two-part role of initial recognition (where flexibility is an advantage) and stabilization of the specific H/HS–antithrombin complex (where rigidity is important) (50). In a manner similar to antithrombin, thrombin also displays quite a few residues with reduced mobility including Arg101 (*R*_g_ = 0.8), Arg165 (*R*_g_ = 0.5) and Lys240 (*R*_g_ = 1.8). These residues are held in place by interaction with neighboring H-bonding groups, e.g., Asp/Gln, or because of a hydrophobic constrain, e.g., Met (Table 2). All three residues contribute to H/HS binding (21,43). Yet, these residues of exosite II do not engineer specificity for thrombin in the manner of antithrombin. This implies that enhanced burial and reduced conformational flexibility are necessary, but not sufficient, for engineering specificity.

Another element that is important for stereospecific recognition is asymmetric organization of points of contact. In principle, all ligand binding sites should be asymmetric. However, GAG binding sites are fundamentally different from traditional, small molecule binding sites [1], [51]. Whereas relatively deep hydrophobic cavities define small molecule binding sites, GAG binding sites are typically shallow. The loss of depth is akin to reduction of three-dimensionality to two, which introduces significant challenges for specificity. A two-dimensional site that displays considerable symmetry is, in effect, a further loss of dimensionality and will encourage multiple, equivalent binding modes and a concurrent loss of specificity. This is especially true if hydrogen bonding, i.e., directionality of interaction, does not contribute significantly to the interaction, as is known to be the case for thrombin [20]. Considering this analysis, exosite II appears to be a fairly symmetric collection of several point charges, whereas the PBS represents an asymmetric pattern of its three important residues, Lys114, Lys125 and Arg129.

A final element that distinguishes the PBS of antithrombin from exosite II of thrombin is the presence of a cavity that is capable of holding tightly bound water molecules. Application of cavity detection tools led to the identification of a bifurcated cavity in the PBS of antithrombin with sizable length (∼20 Å) and depth (∼5 Å) (Figure 6). More importantly, the bifurcated cavity hosts the 6-sulfate of residue D, and 3- and 2- sulfates of residue F, groups known to contribute significantly to pentasaccharide affinity [42]. Further, we computationally localized tightly bound water molecules in this cavity at positions occupied by these sulfates, which suggests a large entropic contribution to specificity, in addition to the enthalpic contribution. The entropic contribution appears to be sufficient large for antithrombin because multiple waters are released. Likewise, the enthalpic contribution also appears to be significant considering that multiple hydrogen bonds are being formed. Thus, although the PBS of antithrombin has been considered as surface-exposed, shallow and electrostatically driven, it is fundamentally different from the many other known GAG-binding sites. Altogether, the PBS of antithrombin is an engineering marvel.

Our analysis did not identify a reasonably sized cavity in exosite II of thrombin. This does not imply that smaller cavities, or depressions, are not present. In fact, we could detect several disjointed, small cavities in exosite II (not shown), but none of these have the size to comfortably host a sulfate group of the H/HS sequence. This implies that, whereas key sulfate groups of the heparin pentasaccharide penetrate into the PBS cavity to form firm ‘hand-shake’ interactions, the interactions of exosite II with H/HS are more superficial and transient.

Our structural analysis suggests that the distinct architecture of the HBSs in antithrombin and thrombin results in distinct roles. The more flexible, surface-exposed residues are primarily responsible for the initial, non-specific recognition of the anionic H/HS ligand, whereas more buried and less conformationally flexible residues are responsible for the recognition of specific H/HS sequences. Stabilization of a specific H/HS–protein complex arises from a significant, complementary, inter-penetration phenomenon that is governed by favorable entropic as well as enthalpic contributions.

These results imply that the specificity of H/HS interaction with a target protein can be elucidated through a rather simple structural analysis. The steps would involve answering questions including: 1) Is there a collection of less surface exposed Arg/Lys? 2) Do these less surface exposed residues exhibit less gyrational mobility? 3) Are there elements of asymmetry in the distribution of these Arg/Lys residues? 4) Does the proposed binding site host a cavity capable of engaging one or more sulfate groups that can replace bound water molecules? If the answers to these questions mimic the answers for antithrombin, the interaction can be expected to be specific. If not, the interaction is likely to be non-specific. We expect that the principles enunciated in this work should help predict/understand fundamental biochemistry of H/HS–protein interactions and facilitate the design of more specific H/HS molecules with therapeutic relevance.
